# Ethanol extract of *Poria cocos* reduces the production of inflammatory mediators by suppressing the NF-kappaB signaling pathway in lipopolysaccharide-stimulated RAW 264.7 macrophages

**DOI:** 10.1186/1472-6882-14-101

**Published:** 2014-03-15

**Authors:** Jin-Woo Jeong, Hye Hyeon Lee, Min Ho Han, Gi-Young Kim, Su Hyun Hong, Cheol Park, Yung Hyun Choi

**Affiliations:** 1 Center for Core Research Facilities, Daegu Gyeongbuk Institute of Science & Technology, Daegu 711-873, Republic of Korea; 2 Department of Biotechnology, College of Natural Resources and Life Science, Dong-A University, Busan 604-714, Republic of Korea; 3 Department of Biochemistry, College of Oriental Medicine, Dongeui University, Busan 614-052, Republic of Korea; 4 Anti-Aging Research Center & Blue-Bio Industry RIC, Dongeui University, Busan 614-714, Republic of Korea; 5 Laboratory of Immunobiology, Department of Marine Life Sciences, Jeju National University, Jeju 690-756, Republic of Korea; 6 Department of Molecular Biology, College of Natural Sciences, Dongeui University, Busan 614-714, Republic of Korea

**Keywords:** *Poria cocos*, RAW 264.7 cells, Anti-inflammation, NF-κB

## Abstract

**Background:**

*Poria cocos* Wolf, a medicinal fungus, is widely used in traditional medicines in East Asian countries owing to its various therapeutic potentials. Although several studies have demonstrated the anti-inflammatory activity of this fungus, its underlying mechanisms have not yet been clearly defined.

**Methods:**

In the present study, we have demonstrated the anti-inflammatory effects of ethanol extract of *P. cocos* (EEPC) in lipopolysaccaride (LPS)-stimulated RAW 264.7 macrophages. As inflammatory parameters, the productions of nitric oxide (NO), prostaglandin E2 (PGE2), interleukin (IL)-1β and tumor necrosis factor (TNF)-α were evaluated. We also examined the EEPC’s effect on the nuclear factor-kappaB (NF-κB) signaling pathway.

**Results:**

Our results indicated that EEPC exhibits a potent inhibitory effect on NO production and inhibits PGE2 release in LPS-induced macrophages without affecting cell viability. EEPC also significantly attenuated LPS-induced secretion of inflammatory cytokines IL-1β and TNF-α. Additionally, LPS-induced expression of inducible NO synthase (iNOS), cyclooxygenase (COX)-2, IL-1β, and TNF-α was decreased by pre-treatment with EEPC at the transcriptional level. Moreover, EEPC clearly inhibited LPS-induced nuclear translocation of NF-κB p65 subunits, which correlated with EEPC’s inhibitory effects on inhibitor kappaB (IκB) degradation. Moreover, EEPC clearly suppressed the LPS-induced DNA-binding activity of NF-κB, as well as the nuclear translocation of the NF-κB p65, which correlated with EEPC’s inhibitory effects on inhibitor kappaB (IκB) degradation.

**Conclusions:**

Taken together, our data indicates that EEPC targets the inflammatory response of macrophages via inhibition of iNOS, COX-2, IL-1β, and TNF-α through inactivation of the NF-κB signaling pathway, supporting the pharmacological basis of *P. cocos* as a traditional herbal medicine for treatment of inflammation and its associated disorders.

## Background

Inflammation is an essential aspect of the host’s response to infection and injury caused by invading pathogens to maintain a healthy state. However, excessive or aberrant inflammation leads to the up-regulation of several kinds of pro-inflammatory enzymes such as nitric oxide synthase (NOS) and cyclooxygenase (COX) as mediators of inflammation in affected inflammatory cells, which contribute to many acute and chronic human diseases [1,2]. NOSs are comprised of three members, including endothelial NOS (eNOS), neuronal NOS (nNOS), and inducible NOS (iNOS), which makes NO from L-arginine, and COX exists as two isozymes, COX-1 and COX-2, converting arachidonic acid into prostaglandins (PGs). Among them, iNOS and COX-2 are highly expressed in response to inflammatory inducers, and are responsible for the production of a huge amount of NO and prostaglandin E2 (PGE2), respectively [3,4]. The inflammatory response is also well characterized by the abundant production of pro-inflammatory cytokines, such as interleukin-1β (IL-1β) and tumor necrotic factor-α (TNF-α). They are also mainly produced in activated macrophages by inflammatory inducers. Overproduction of IL-1β increases the expression of adhesion factors on endothelial cells to enable transmigration of leukocytes, and is associated with hyperalgesia and fever [5,6]. In particular, TNF-α is important for stimulating the secretion of other inflammatory cytokines, which, in turn, causes many clinical problems associated with autoimmune disorders [7,8]. Key pro-inflammatory stimuli including bacterial lipopolysaccharide (LPS), mitogens, and cytokines modulate their effects by inducing the activation of nuclear factor-kappaB (NF-κB), which regulates the expression of many genes involved in immune and inflammatory responses [9,10]. For this reason, inhibition of pro-inflammatory mediators and cytokines by these NF-κB response genes has been proposed to be a good approach for the treatment of various inflammatory diseases [11,12].

*Poria cocos* Wolf is a medicinal mushroom in the *Polyporaceae* family that grows on the roots of old, dead pine trees. The dried sclerotia of *P. cocos* has frequently been used as a tonic to benefit the internal organs and is prescribed as one of the chief ingredients in compound prescriptions in traditional Oriental medicine [13-15]. In particular, this fungus is widely used in traditional medicine to treat chronic gastritis, acute gastroenteric catarrh, gastric atony, oedema, nephrosis, dizziness, nausea, and emesis [16,17]. Many previous studies have indicated that its extracts and components have a variety of biological activities such as anti-fungal and anti-bacterial [18], antioxidant [19-21], neuroprotective [22], anti-hypertonic [23], anti-inflammatory [17,24-26], anti-angiogenic [27,28], and anti-cancer effects [29,30]. However, the claimed benefits and their action mechanisms are not fully understood. In this study, as a part of our on-going screening program to evaluate the anti-inflammatory potentials of medicinal mushrooms, we investigated the anti-inflammatory properties of an ethanol extract of *P. cocos* (EEPC) and the responsible underlying molecular mechanisms involved in an LPS-stimulated RAW 264.7 murine macrophage model. We found that EEPC down-regulated the production of pro-inflammatory mediators (NO and PGE2) as well as pro-inflammatory cytokines (IL-1β and TNF-α) by suppressing the NF-κB signaling pathway.

## Methods

### Materials

LPS, Griess reagent, Tween 20, 3-(4,5-dimethylthiazol-2-yl)-2,5-diphenyltetrazolium bromide (MTT), dimethyl sulfoxide (DMSO), and 4,6-diamidino-2-phenyllindile (DAPI) were purchased from Sigma-Aldrich Chemical Co. (St. Louis, MO, USA). Antibodies against iNOS, COX-2, NF-κB p65, inhibitor kappaB (IκB), nucleolin and actin were purchased from Santa Cruz Biotechnology (Santa Cruz, CA, USA). The peroxidase-labeled donkey anti-rabbit immunoglobulin, peroxidase-labeled sheep anti-mouse immunoglobulin and enhanced chemiluminescence (ECL) detection kit were purchased from Amersham Corp. (Arlington Heights, IL, USA). Dulbecco’s modified Eagle’s minimum essential medium (DMEM) containing l-glutamine (200 mg/L), fetal bovine serum (FBS), penicillin, and streptomycin were obtained from Gibco-BRL (Grand Island, NY, USA). The enzyme-linked immunosorbent assay (ELISA) kits for PGE2, TNF-α, and IL-1β were obtained from R&D Systems (Minneapolis, MN, USA). COX-2, iNOS, TNF-α, IL-6, and GAPDH oligonucleotide primers were purchased from Bioneer (Seoul, Korea). Fluorescein isothiocyanate (FITC)-conjugated donkey anti-rabbit IgG and Fluoromount-G were obtained from Jackson ImmunoResearch Laboratories Inc. (West Grove, PA, USA) and Southern Biotechnology Associates Inc. (Birmingham, AL, USA), respectively. All other chemicals were purchased from Sigma-Aldrich.

### Preparation of the ethanol extract of *P. cocos* (EEPC)

The dried sclerotium of *P. cocos* were supplied by Dongeui University Oriental Hospital (Busan, Republic of Korea) and authenticated by Professor S.H. Hong, Department of Biochemistry, Dongeui University College of Oriental Medicine. A voucher specimen (accession number DEU-24) was deposited at the Natural Resource Bank of Dongeui University College of Oriental Medicine. To prepare the EEPC, the dried sclerotium of *P. cocos* was ground into powder and extracted twice with 10 volumes of 80% ethanol at 85-90˚C in a reflux condenser for 3 h. After being filtered through a 0.2-μm filter, the extract was concentrated and lyophilized by vacuum evaporation at 60˚C. The solid form of the extract was dissolved in DMSO prior to the experiment.

### Cell culture and MTT assay

The RAW 264.7 macrophage cell line was obtained from American Type Culture Collections (Manassas, VA, USA) and cultured at 37°C in 5% CO_2_ in DMEM medium supplemented with 10% FBS, 100 units/ml penicillin, and 100 μg/ml streptomycin in the presence or absence of EEPC. The cell viability was measured using a MTT assay. Briefly, RAW 264.7 cells (5?×?10^5^ cells/ml) were treated with the indicated concentrations of EEPC or LPS (0.5 μg/ml) alone, or pre-treated with different concentrations of EEPC for 1 h before LPS treatment. After 24 h, the medium was removed and the cells were incubated with 0.5 mg/ml of MTT solution for 2 h. And then, the supernatant was discarded and the formazan blue, which was formed in the cells, was dissolved with DMSO. The optical density was measured at 540 nm with a microplate reader (Dynatech Laboratories, Chantilly VA, USA).

### Nitrite determination

The nitrite accumulated in culture medium was measured as an indicator of NO production based on the Griess reaction. Briefly, 100 μl of cell culture medium was collected at the end of culture, mixed with an equal volume of Griess reagent, incubated at room temperature. After 10 min, the absorbance at 540 nm was measured using an ELISA plate reader at 540 nm. The concentration of nitrite was calculated from a standard curve drawn with known concentrations of sodium nitrite dissolved in DMEM [31].

### Determination of PGE2, TNF-α, and IL-1β production

RAW 264.7 macrophages were pre-treated with EEPC for 1 h and then stimulated with LPS (0.5 μg/ml) for 24 h. PGE2, TNF-α, and IL-1β levels in macrophage culture media were quantified using ELISA kits according to the manufacturer’s instructions [32].

### RNA isolation and reverse transcriptase polymerase chain reaction (RT-PCR) assay

After the removal of supernatants from cells cultured in the presence of EEPC alone or in combination with LPS for 24 h, total RNA was isolated using TRIzol reagent (Invitrogen Co., Carlsbad, CA, USA) according to the manufacturer’s instructions. From each sample, 2 μg of total RNA was reverse transcribed to single-stranded cDNA by M-MLV reverse transcriptase (Promega, Madison, WI). Then PCR analyses were performed on the aliquots of the cDNA preparations to detect COX-2, iNOS, TNF-α, and IL-1β gene expression. The iNOS, COX-2, IL-1β, and TNF-α genes were amplified from the cDNA using PCR. The PCR primers were as follows: mouse iNOS (5′-ATG TCC GAA GCA AAC ATC AC-3′ and 5′-TAA TGT CCA GGA AGT AGG TG-3′), COX-2 (5′-CAG CAA ATC CTT GCT GTT CC-3′ and 5′-TGG GCA AAG AAT GCA AAC ATC-3′), IL-1β. (5′-ATG GCA ACT GTT CCT GAA CTC AAC T-3′ and 5′-TTT CCT TTC TTA GAT ATG GAC AGG AC-3′), and TNF-α (5′-ATG AGC ACA GAA AGC ATG ATC-3′ and 5′-TAC AGG CTT GTC ACT CGA ATT-3′). After amplification, the PCR products were electrophoresed in 1% agarose gels and visualized by ethidium bromide (EtBr) staining and ultra violet (UV) irradiation. In a parallel experiment, glyceraldehyde-3-phosphate dehydrogenase (GAPDH) was used as an internal control.

### Protein extraction and Western blot analysis

After 24 h treatment as described above, the total proteins of RAW 264.7 cells were directly prepared in lysis buffer (0.5% Triton, 50 mM β-glycerophosphate (pH 7.2), 0.1 mM sodium vanadate, 2 mM MgCl_2_, 1 mM EGTA, 1 mM dithiothreitol, 2 μg/mL leupeptin, 0.1 mM phenylmethylsulfonyl urea, and 4 μg/mL aprotinin). In a parallel experiment, nuclear and cytosloic proteins were prepared using nuclear extraction reagents (Pierce, Rockford, IL, USA) according to the manufacturer’s protocol. The protein concentration in the cell lysate was determined using detergent-compatible protein assay from Bio-Rad (Hercules, CA, USA). Equal amounts of protein were resolved by sodium dodecyl sulfate (SDS)-polyacrylamide gel electrophoresis and transferred to nitrocellulose membrane (Schleicher & Schuell, Keene, NH, USA). Subsequently, the membranes were blocked in Tris-buffered saline (10 mM Tris-Cl, pH 7.4) containing 0.5% Tween 20 and 5% nonfat dry milk for 1 h. After incubation with the appropriate primary antibodies for 1 h, the membranes were incubated for 1 h at room temperature with secondary antibodies conjugated to horseradish peroxidase. The immunoreactive bands were detected by ECL solution.

### Electrophoretic mobility assay (EMSA)

EMSA was performed with the nuclear extract. Synthetic complementary NF-κB (5′-AGT TGA GGG GAC TTT CCC AGG C-3′) binding oligonucleotides (Santa Cruz Biotechnology) were 3′-biotinylated using the biotin 3′-end DNA labeling kit (Pierce) according to the manufacturer’s instructions, and annealed for 30 min at room temperature. Assays were loaded onto native 4% polyacrylamide gels pre-electrophoresed for 60 min in 0.5× Tris borate/EDTA before being transferred onto a positively charged nylon membrane (HybondTM-N^+^) in 0.5 × Tris borate/EDTA at 100 V for 30 min. The transferred DNAs were cross-linked to the membrane at 120 mJ/cm2. Horseradish peroxidase-conjugated streptavidin was used according to the manufacturer’s instructions to detect the transferred DNA.

### Immunofluorescence staining

The NF-κB p65 nuclear localization was detected by immunofluorescence assays using a fluorescence microscope. For this study, RAW 264.7 cells were cultured directly on glass coverslips in 24-well plates for 24 h. After stimulation with LPS in the presence or absence of EEPC, the cells were fixed with 4% paraformaldehyde in PBS, permeabilized with 0.2% triton X-100 in PBS, and blocked with 1.5% normal donkey serum. Polyclonal antibodies against anti-NF-κB p65 (1 μg/well) were applied for 1 h followed by an 1 h incubation with FITC-conjugated donkey anti-rabbit IgG. The position of the cell nucleus was determined with DAPI. After washing with PBS, the coverslips were mounted in Fluoromount-G, and the fluorescence was visualized using a fluorescence microscope (Carl Zeiss, Germany) [33].

#### Statistical analysis

All values are presented as mean ± standard deviation (SD). We assessed comparisons between groups by one-way analysis of variance (Dunnett’s *t*-test) and Student’s *t*-test. P values ≤ 0.05 were considered statistically significant.

## Results

### EEPC inhibits LPS-induced NO and PGE2 production in RAW 264.7 macrophages

**Figure 1:**
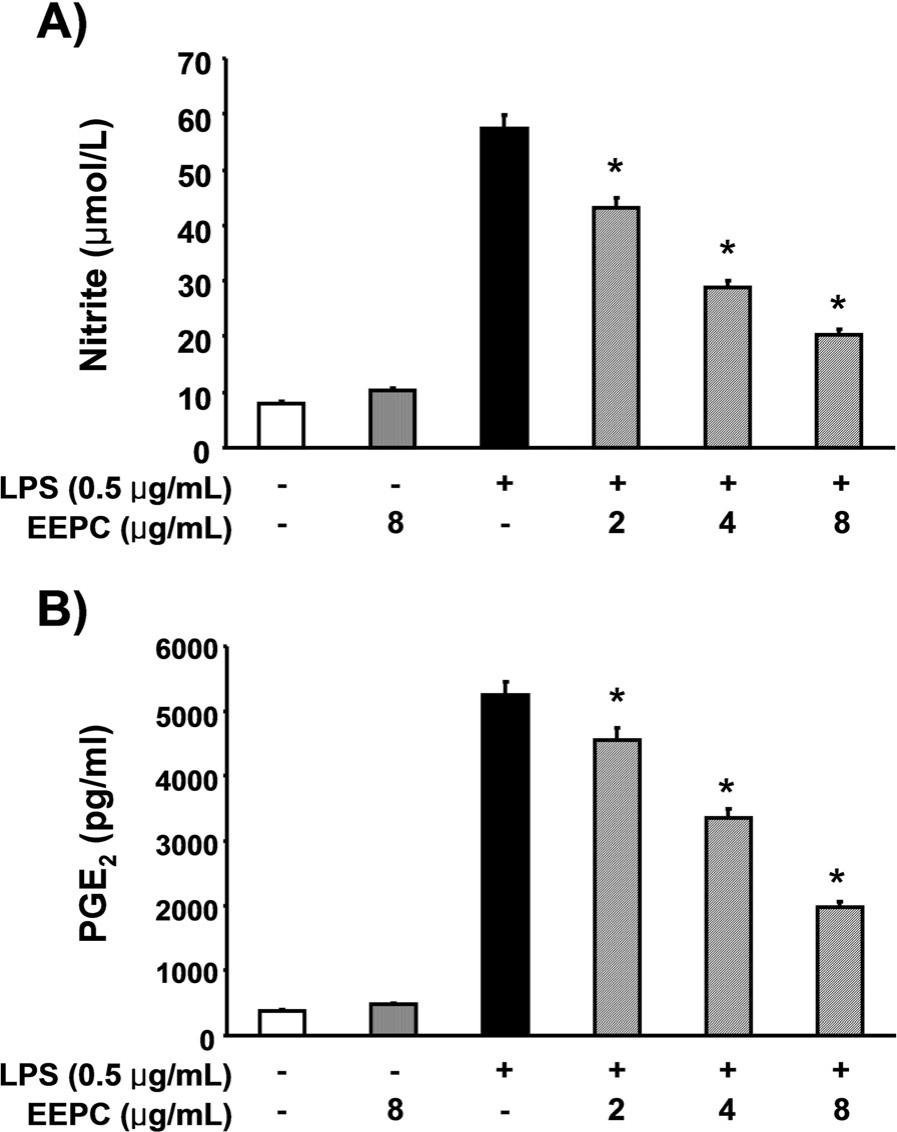
**Inhibition of NO and PGE2 production by EEPC pre-treatment in LPS-stimulated RAW 264.7 macrophages.** Cells were pre-treated with different concentrations of EEPC for 1 h, LPS (0.5 μg/mL) was then added, and cells were incubated for 24 h. The culture supernatants were subject to the nitrite assay **(A)** and to a PGE2 immunoassay **(B)**. Values represent the mean ± SD of three independent experiments. We assessed differences between mean values by the Student’s *t*-test. *P < 0.05 indicates significant differences from the LPS-treated group.

RAW 264.7 cells were stimulated with LPS for 24 h after being pre-treated with various concentrations of EEPC for 1 h, and cell culture media were collected, and NO levels were quantified initially using the Griess reaction. As shown in Figure 1A, when LPS was added to RAW 264.7 cells, NO production was increased dramatically, however, EEPC suppressed LPS-induced NO production in a concentration-dependent manner. Moreover, the PGE2 assay revealed that EEPC significantly attenuated LPS-induced PGE2 production (Figure 1B).

### EEPC down-regulates LPS-induced iNOS and COX-2 expression in RAW 264.7 macrophages

**Figure 2:**
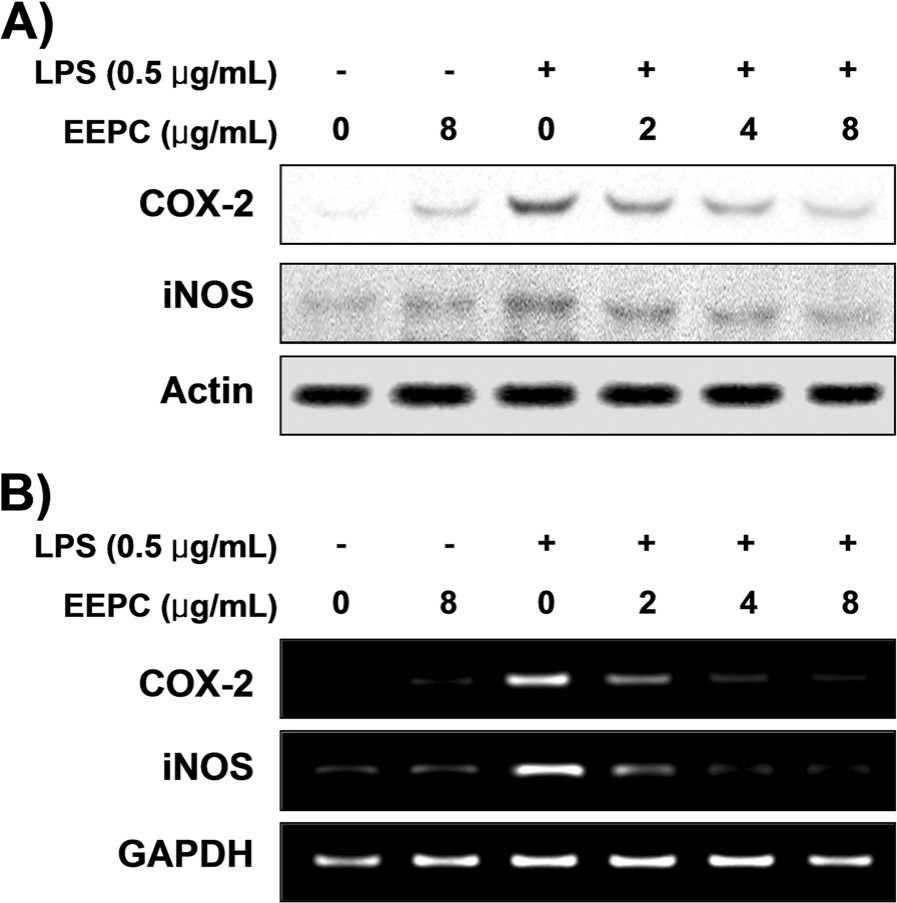
**Down-regulation of LPS-induced iNOS and COX-2 protein and mRNA expression by EEPC pre-treatment in RAW 264.7 macrophages. (A)** Cells were pre-treated with different concentrations of EEPC for 1 h, then with LPS (0.5 μg/ml), and incubated for 24 h. Total cellular proteins (30 μg) were resolved by SDS-polyacrylamide gel electrophoresis, transferred to nitrocellulose membranes, and detected with anti-COX-2 and iNOS antibodies, and an ECL detection system. **(B)** After LPS treatment for 6 h, total RNA was prepared for the RT-PCR analysis of COX-2 and iNOS gene expression. The amplified PCR products were run in a 1% agarose gel and visualized by EtBr staining. Actin and GAPDH were used as internal controls for the Western blot and RT-PCR assays, respectively.

To determine the inhibitory mechanism of EEPC on NO and PGE2 production from LPS-activated RAW 264.7 cells, we examined the expression levels of iNOS and COX-2 protein and mRNA by Western blot and RT-PCR analyses. In response to LPS, the protein levels of iNOS and COX-2 were significantly induced; however, pre-treatment with EEPC dramatically inhibited these up-regulations in a concentration-dependent manner (Figure 2A). Furthermore, pre-treatment with EEPC markedly suppressed mRNA expression of iNOS and COX-2 in the same manner as for their protein expression (Figure 2B). These results indicate that the reductions in the expression of iNOS and COX-2 at the transcriptional levels contributed to the inhibitory effect of EEPC on LPS-induced NO and PGE2 production.

### EEPC reduces the production of pro-inflammatory cytokines in LPS-stimulated RAW 264.7 macrophages

**Figure 3:**
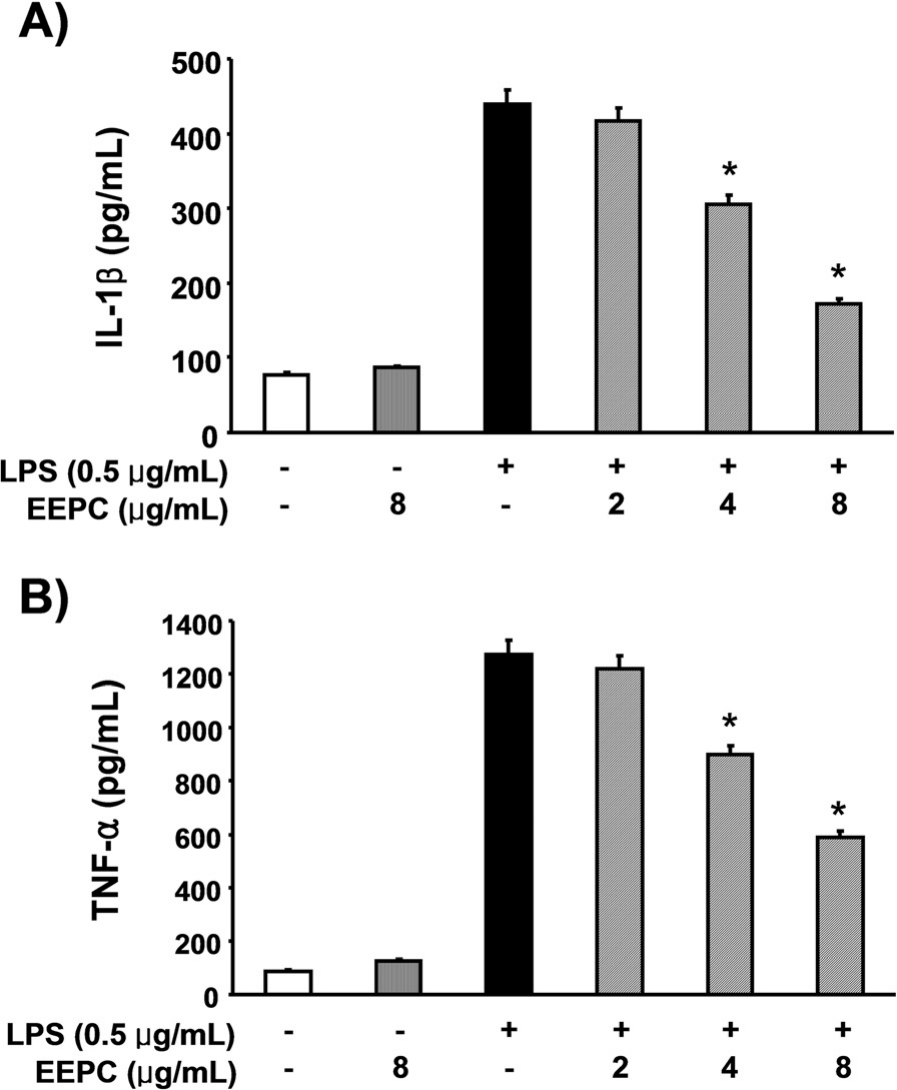
**Effects of EEPC on LPS-induced IL-1β and TNF-α release in RAW 264.7 macrophages.** Cells were pre-treated with the indicated concentrations of EEPC 1 h prior to incubation with LPS (0.5 μg/ml). After incubation for 24 h, the levels of IL-1β **(A)** and TNF-α **(B)** present in the supernatants were measured using ELISA kits. The values shown here are means ± SD of three independent experiments. * *P* < 0.05 indicates a significant difference from the value obtained for cells treated with LPS in the absence of EEPC.

**Figure 4:**
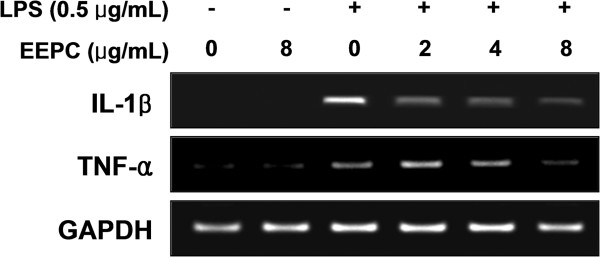
**Effects of EEPC on LPS-induced IL-1β and TNF-α expression in RAW 264.7 macrophages.** The cells were pre-treated with various concentrations of EEPC for 1 h before LPS treatment (0.5 μg/ml), and total RNA was isolated at 6 h after LPS treatment. The levels of IL-1β and TNF-α mRNA were determined using RT-PCR. GAPDH was used as an internal control.

Since EEPC was revealed as a potent inhibitor of the pro-inflammatory mediators, we further investigated its effect on pro-inflammatory cytokine release by ELISA. The results obtained showed that treatment of RAW 264.7 cells with LPS alone resulted in a significant increase in production of IL-1β compared to that generated under control conditions (Figure 3A). However, pre-treatment with EEPC considerably inhibited LPS induction of IL-1β in a concentration-dependent manner. Under these conditions, pre-treatment with EEPC also reduced TNF-α production dramatically (Figure 3B). Furthermore, the RT-PCR results showed that non-activated or EEPC-alone treated RAW 264.7 cells did not express any detectable levels of IL-1β and TNF-α mRNA; however, EEPC significantly attenuated LPS-induced mRNA levels of these cytokines (Figure 4).

### EEPC inhibits the nuclear translocation of NF-κB in LPS-stimulated RAW 264.7 macrophages

**Figure 5:**
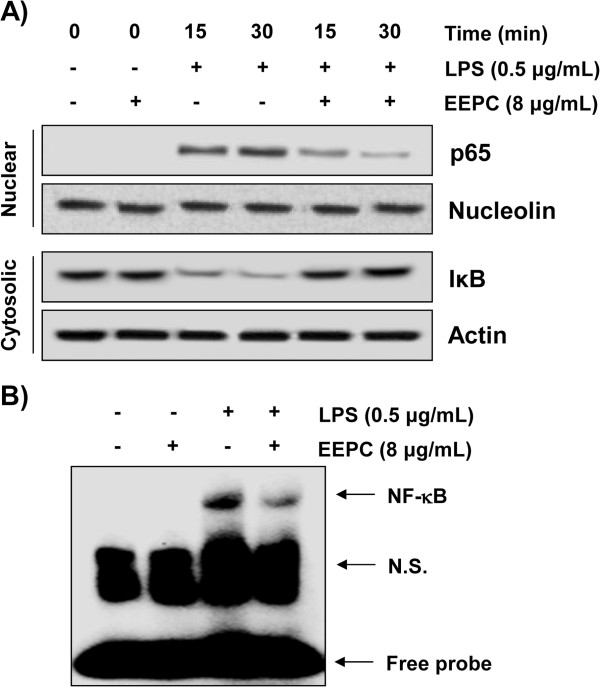
**Inhibition of LPS-induced nuclear accumulation of NF-κB p65 and degradation of IκB-α by EEPC pre-treatment in RAW 264.7 macrophages.** Cells were treated with 8 μg/ml EEPC for 1 h before LPS treatment (0.5 μg/ml) for the indicated times. **(A)** Nuclear and cytosolic proteins were subjected to 10% SDS-polyacrylamide gels followed by Western blotting using anti-NF-κB p65 and anti-IκB-α antibodies. Nucleolin and actin were used as internal controls for the nuclear and cytosolic fractions, respectively. **(B)** Cells were pre-incubated with EEPC 1 h before stimulation with LPS for 30 min. Nuclear extracts were then assayed for NF-κB activity by EMSA (N.S.; non specific).

**Figure 6:**
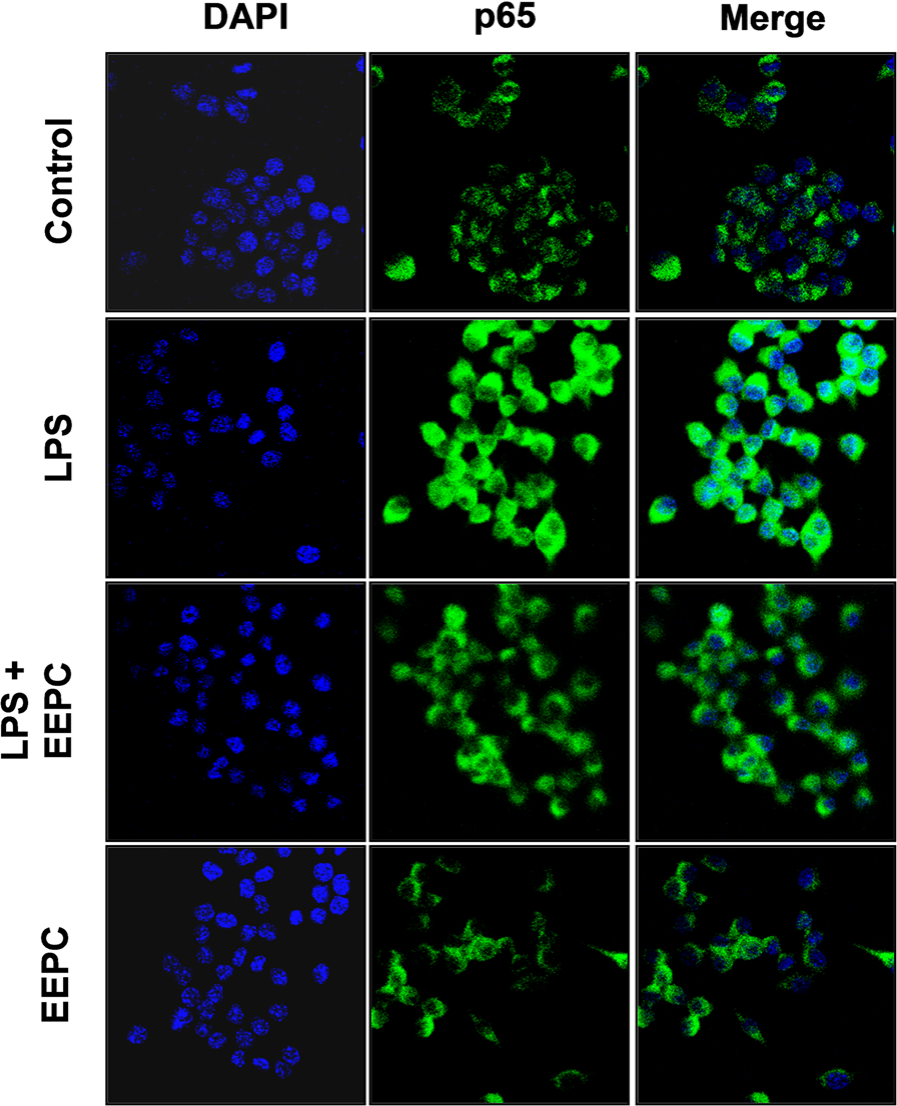
**Effects of EEPC on LPS-induced nuclear translocation of NF-κB p65 in RAW 264.7 macrophages.** Cells were pre-treated with 8 μg/ml EEPC for 1 h, LPS (0.5 μg/mL) was then added, and cells were incubated for 30 min. Localization of NF-κB p65 was visualized with fluorescence microscopy after immunofluorescence staining with NF-κB p65 antibody (green). Cells were stained with DAPI for visualization of the nuclei (blue). A representative sample of three independent experiments is shown.

To further characterize the mechanism underlying the anti-inflammatory effects of EEPC, we assessed the NF-κB signaling pathway, which is critical in the activation of pro-inflammatory enzymes and cytokines. Immunoblotting results in Figure 5A showed that the amount of NF-κB p65 in the nucleus was rapidly increased after exposure to LPS alone, concomitantly with a degradation in IκB-α. However, pre-treatments with EEPC markedly reduced the LPS-induced nuclear accumulation of NF-κB p65. In parallel with the inhibitory effect of EEPC, LPS-induced IκB-α degradation was obviously blocked by pre-treatment with EEPC. EMSA also showed that treatment with LPS causes an increase in NF-κB DNA-binding activity at 30 min, while pretreatment of the cells with EEPC for 1 h resulted in a significant reduction in the DNA-binding activity of NF-κB (Figure 5B). In addition, the immunofluorescence images revealed that NF-κB p65 was normally sequestered in the cytoplasm, and nuclear translocation of NF-κB p65 was not observed in the cells after treatment with EEPC alone in the absence of LPS stimulation (Figure 6). However, the nuclear localization of NF-κB p65 in RAW 264.7 cells was significantly induced after stimulation with LPS, which was completely abolished after pre-treating the cells with EEPC. The findings indicate that the inactivation of the NF-κB signaling pathway was involved in the anti-inflammatory effect of EEPC in LPS-stimulated RAW 264.7 cells.

### Effects of EEPC on the viability of RAW264.7 macrophages

**Figure 7:**
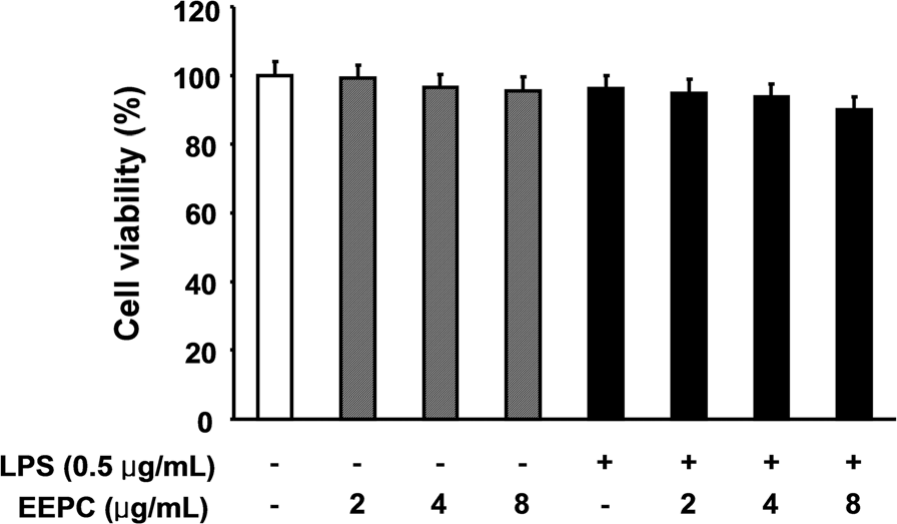
**Effect of EEPC on the cell viability of RAW 264.7 macrophages.** Cells were treated with the various concentrations of EEPC for 1 h and then the cells were stimulated with 0.5 μg/mL LPS. After 24 h, cytotoxicity was determined by measuring the absorbance at 450 nm after MTT reagent addition and the results are expressed as the percentage of surviving cells over control cells (no addition of EEPC and LPS). The values shown here are means ± SD of three independent experiments.

To examine whether EEPC is cytotoxic to RAW 264.7 cells, the cells were exposed to various concentrations of EEPC for 24 h in the presence or absence of LPS, and cell viability was then measured by the MTT assay. Results showed that, within our tested concentrations, no EEPC cytotoxic effect was observed (Figure 7). These results clearly indicated that the anti-inflammatory activity of EEPC in LPS-stimulated RAW 264.7 macrophages was not due to its cytotoxicity.

## Discussion

Although the dried sclerotia of *P. cocos*, a well-known medicinal fungus, is widely used in traditional Oriental medicine owing to its multiple beneficial potentials, the molecular targets and the mechanisms underlying its anti-inflammatory activities are still unclear to date. Inhibitors of pro-inflammatory mediators and cytokines have been considered as candidates for anti-inflammatory agents. In the present study, we first demonstrated that EEPC reduced LPS-induced NO and PGE2 production in RAW 264.7 macrophages through down-regulation of iNOS and COX-2 at both the protein and mRNA levels, which suggests that EEPC acts at the transcriptional level. EEPC also reduced the LPS-induced production and mRNA expression of the pro-inflammatory cytokines, IL-1β and TNF-α. Furthermore, these effects were not due to the cytotoxicity of EEPC and were mediated through the inhibition of the nuclear translocation of NF-κB.

Among the pro-inflammatory mediators released by macrophages, NO and PGE2 have been implicated as the main cytotoxic mediators participating in the innate response in mammals. Although NO generation by macrophages is a part of the human immune response, activated macrophages following bacterial infection or inflammatory responses generate a large amount of NO from arginine and oxygen through transcriptional activation of iNOS. Furthermore, excessive release of NO by activated macrophages is correlated with the progression of inflammation-associated disorders [2,34]. There is mounting evidence for the expression of COX-2, a key enzyme involved in the conversion of arachidonic acid to PGE2, being up-regulated in activated macrophages. In addition, the activation of COX-2 is associated with cytotoxicity at the site of the inflammation, because inhibition of COX-2 induction and/or activity reduces the progression of inflammatory disorders [1,3]. Thus, down-regulators of these pro-inflammatory mediators have been considered as potential candidates for anti-inflammatory agents to alleviate the progression of inflammatory diseases such as atherosclerosis, chronic hepatitis, pulmonary fibrosis and rheumatoid arthritis caused by the activation of macrophages. Our results clearly demonstrated that EEPC inhibited NO and PGE2 production via the suppression of iNOS and COX-2 expression, respectively, which appears to be due to the transcriptional suppression of these genes (Figures 1 and 2).

The release of pro-inflammatory cytokines is elevated predominantly by activated macrophages during pathogen infection and they are involved in the up-regulation of inflammatory reactions. Of these cytokines, IL-1β is a potent activator of immune responses directed against bacterial infections. Abnormal IL-1β release changes the conditions toward a pathological microenvironment, resulting either in chronic inflammation or in the absence of a proper immune surveillance against infections, respectively [6,35]. In addition, inflammation is often associated with the over-expression of TNF-α, which is a multi-functional cytokine involved in the signaling pathways implicated in inflammation, immunity, cell survival, and even tumorigenesis. This cytokine exhibits its pro-inflammatory activity by regulating several intercellular and vascular cell-adhesion molecules, resulting in the recruitment of leukocytes to sites of inflammation [7,8]. Moreover, the production of TNF-α and IL-1β is crucially required for the synergistic induction of NO and PGE2 production in LPS-stimulated macrophages [36,37]. Such findings suggest that the inhibition of these cytokines’ production could be a useful approach as a treatment strategy for various inflammatory diseases. In the current investigation, the concentrations of TNF-α and IL-1β were markedly increased after treatment with LPS in RAW 264.7 macrophages; while pre-treatment with EEPC significantly inhibited this effect in a concentration-dependent manner (Figure 3). We also found that the suppressive effect of EEPC on LPS-induced production of TNF-α and IL-1β was mediated at the transcription level (Figure 4). These findings provide evidence that EEPC possesses potentially useful anti-inflammatory activities.

Of the several transcriptional factors activated by inflammatory responses during bacterial infections, NF-κB plays a critical role in several signal transduction pathways. Hence, agents that are able to inhibit NF-κB transcriptional regulation and modulate the inflammatory response may have therapeutic use, and there is a growing interest among researchers in targeting the NF-κB signaling pathway in the fight against inflammation [11,12]. In the absence of stimuli, in most cells, NF-κB is associated with inhibitor proteins, IκBs, and as a result, NF-κB is retained in the cytoplasm. However, when the cells are stimulated with pro-inflammatory stimuli, IκBs are rapidly phosphorylated, degraded, and thereby dissociated from NF-κB. The resulting free NF-κB is then translocated into the nucleus, where it binds to the κB elements and induces the transcription of genes encoding pro-inflammatory mediators and cytokines [38,39]. Thus, we presumed that the above inhibitory effect of EEPC on the LPS-induced overproduction of pro-inflammatory mediators and cytokines involves the NF-κB signaling pathway; we next measured the effects of EEPC on LPS-induced-NF-κB nuclear translocation. Western blotting, EMSA and immunofluorescence results revealed that EEPC inhibited the LPS-induced degradation of IκB-α and the subsequent translocation of the p65 subunit of NF-κB from the cytosol to the nucleus, and markedly attenuated LPS-induced NF-κB binding activity (Figures 5 and 6). These data indicated that the anti-inflammatory effect of EEPC might be the result of the inhibition of the degradation of IκB, and then a reduction in NF-κB p65 translocation to the nucleus.

## Conclusions

In conclusion, our study demonstrated that EEPC is a potent inhibitor of pro-inflammatory mediators and cytokines in an LPS-stimulated RAW 264.7 murine macrophage cell model via down-regulating the NF-κB signaling pathway to attenuate their corresponding gene activations. Although further studies are still needed, these findings provide a partial molecular explanation for the anti-inflammatory properties of EEPC.

## Competing interests

The authors declare that they have no competing interests.

## Authors’ contributions

JWJ and HHL carried out the studies and drafted the manuscript. MHH and GYK participated in the design of the study data analyses and manuscript. CP and SHH conceived of the study, and participated in its design and coordination and helped to draft the manuscript. All authors read and approved the final manuscript.

## Pre-publication history

The pre-publication history for this paper can be accessed here:

http://www.biomedcentral.com/1472-6882/14/101/prepub
